# Calcifediol: Why, When, How Much?

**DOI:** 10.3390/ph16050637

**Published:** 2023-04-22

**Authors:** Simone Donati, Francesca Marini, Francesca Giusti, Gaia Palmini, Cinzia Aurilia, Irene Falsetti, Teresa Iantomasi, Maria Luisa Brandi

**Affiliations:** 1 Department of Experimental and Clinical Biomedical Sciences, University of Florence, 50139 Florence, Italy; 2 Fondazione Italiana Ricerca Sulle Malattie dell’Osso (FIRMO Onlus), 50129 Florence, Italy

**Keywords:** vitamin D, calcifediol, 25(OH)D_3_, hypovitaminosis D, vitamin D deficiency, non-genomic effects, vitamin D receptor, membrane-associated rapid response to steroid

## Abstract

Vitamin D deficiency is a constantly growing health problem worldwide. Adults affected with hypovitaminosis D could experience negative consequences on their musculoskeletal system and extra-skeletal health. In fact, an optimal vitamin D status is essential to ensure the correct bone, calcium, and phosphate homeostasis. To improve vitamin D status, it is important to not only increase the intake of food fortified with vitamin D, but also to administer vitamin D supplementation when required. Vitamin D3 (cholecalciferol) is the most widely used supplement. In recent years, the administration of calcifediol (25(OH)D3), the direct precursor of the biologically active form of vitamin D3, as oral vitamin D supplementation has progressively grown. Here, we report the potential medical benefits of some peculiar biological actions of calcifediol, discussing the possible specific clinical scenarios in which the oral intake of calcifediol could be most effective to restore the correct serum levels of 25(OH)D3. In summary, the aim of this review is to provide insights into calcifediol-related rapid non-genomic responses and the possible use of this vitamin D metabolite as a supplement for the treatment of people with a higher risk of hypovitaminosis D.

## 1. Introduction

Vitamin D is an essential micronutrient that is enzymatically converted into a multifunctional active secosteroid hormone, calcitriol (1α,25(OH)_2_D_3_), fundamental for human health over the whole lifetime [1].

The human population currently has a high prevalence of modest-to-severe vitamin D deficiency, which negatively impacts the correct functioning of the musculoskeletal and extra-skeletal systems. This makes hypovitaminosis D a growing and strongly felt worldwide health problem [2]. 

The main source of vitamin D is endogenous production in the epidermis of the skin following exposure to sunlight. In particular, solar ultraviolet B (UVB) photon irradiation (around 290 to 310 nm) is absorbed by 7-dehydrocholesterol (7-DHC) to produce pre-vitamin D_3_ which undergoes a temperature-sensitive rearrangement of three double bonds to form vitamin D_3_ or cholecalciferol (vitD_3_) [3,4,5]. However, it has to be taken into consideration that sun exposure should be implemented in the middle of the day (approximately 12 pm–2 pm) when the incidence of UVB rays is perpendicular; however, there is also a higher risk of UVB-mediated mutagenic DNA damage that could lead to skin cancer development (such as melanoma) [6]. Therefore, it is difficult to find a balance between sufficient exposure to UVB rays to produce an adequate amount of vitamin D for bone and general health and the increased risks of skin damage. Moreover, the capability of sunlight to produce pre-vitamin D_3_ progressively diminishes from the equator, and it is also influenced by other factors, such as climatic conditions, skin color, cultural habits, sun-avoidance practices, and aging, because in older adults the capacity of cutaneous vitamin D synthesis can be decreased by up to 75% [7].

The diet represents another source of vitamin D_3_, which can be taken in small quantities in only a few foods, such as fortified dairy products and fish oils, or as the vitamin D_2_ isoform from vegetables [8].

**Figure 1:**
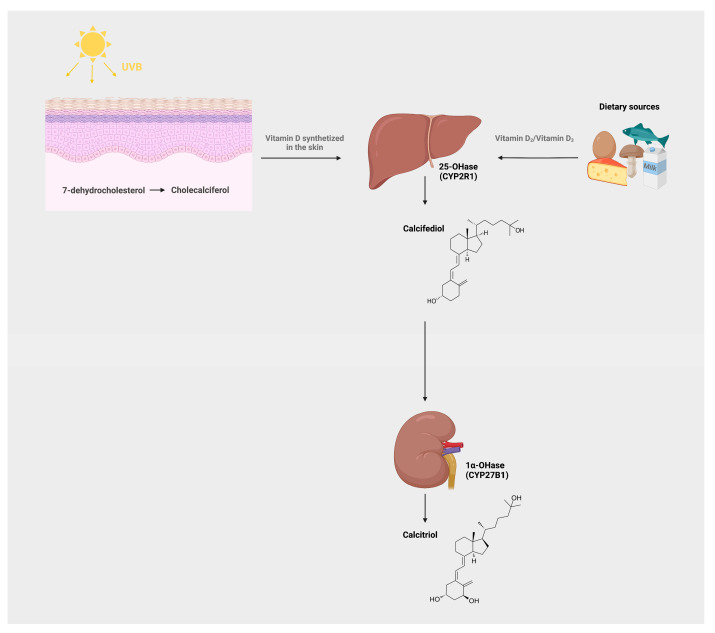
Vitamin D metabolic pathway.

Once produced or orally taken, vitamin D_3_ is metabolically inactive and it is transported in the blood bound to albumin or vitamin D-binding protein (DBP) to the liver, where it undergoes a first hydroxylation step at the C25-position, by the 25-hydroxylase, to produce calcifediol or calcidiol (25(OH)D_3_), the major circulating form of vitamin D (Figure 1). When compared to the other vitamin D metabolites, the half-life of 25(OH)D_3_ varies from 20 to 24 days, which is the highest compared to the other vitamin D metabolites [9,10]. The 25(OH)D_3_ is then transported by DBP primarily to the proximal tubules of the kidney, where a cargo receptor complex composed of megalin, a 600 KDa transmembrane protein, and cubulin can mediate the uptake of complexes of DPB and 25(OH)D_3_ in the tubular epithelial cells from the luminal site by endocytic internalization [11,12]. Here, 25(OH)D_3_ undergoes a second hydroxylation step at the C1 alpha position, by the mitochondrial 1α-hydroxylase, to obtain the biologically active form of vitamin D_3_, 1α,25(OH)_2_D_3_ [13].

It has been reported that 25(OH)D_3-_1α-hydroxylase (also known as CYP27B1) is expressed in several cells other than renal tubes, including keratinocytes, brain glial cells, monocytes, and parathyroid cells. However, under physiological conditions, the kidney is the only organ that produces and releases the hormone calcitriol into the circulation, while all the other cells and tissues secrete 1α,25(OH)_2_D_3_ only in a paracrine–autocrine manner [6]. 

The kidney-produced active vitamin D_3_ reaches different target tissue cells via the circulation, including bone, kidney, and intestine, where it exerts its physiological key functions, such as calcium and phosphate intestinal absorption and the regulation of bone metabolism. Since the isolation and identification of the 1α,25(OH)_2_D_3_ in 1971, additional non-calcemic and non-osteogenic roles of vitamin D have been also reported [14]. 

Calcitriol is a calciotropic hormone that functions as a steroid through its binding to the intracellular vitamin D receptor (VDR) [15], a member of the steroid nuclear receptor superfamily, which comprises thyroid hormones, retinoic acid, sex hormones, and adrenal steroids, resulting in the modulation (activation or suppression) of gene expression [13,16,17,18,19]. Interestingly, calcifediol was also demonstrated to be able to bind and activate VDR, even with a 50 times lower affinity and activating strength than the active vitamin D [20]. The *VDR* gene, comprising eight coding exons, is evolutionarily conserved among birds, fish, and mammals, encoding a protein of 427 amino acids [21,22]. VDR contains a conserved NH_2_-terminal DNA-binding domain (DBD) and a more variable COOH-terminal ligand-binding domain (LBD) [23]. The 1α,25(OH)_2_D_3_ binding induces a conformational change that prevents the repressor protein from binding and facilitates receptor homodimerization to form the VDR:VDR complex or the interaction of VDR with retinoid X receptor (RXR) to form the heterodimeric complex VDR:RXR [24]. Both homo- and heterodimeric complexes are able to bind to repeated sequences positioned in the proximity of transcription start sites of target genes, known as vitamin-D-responsive elements (VDREs), thereby modulating positively or negatively the transcription of genes whose protein products are involved in the control of calcium homeostasis (i.e., cytochrome P450 family (CYP450) 24 (CYP24), type I collagen (COL1A1), alkaline phosphatase (ALP), parathyroid hormone (PTH), PTH-related peptide, transient receptor potential vanilloid type family member 6 (TRPV6), osteopontin, and osteocalcin (bone gamma-carboxyglutamate protein, BGLAP)) [13,16,17,18,19]. 

Recently, both calcitriol and calcifediol have been demonstrated to be able to exert rapid non-genomic effects on target cells, mediated by their binding to a membrane isoform of VDR, ascertaining a more complex mechanism responsible for the broad spectrum of vitamin D actions [23].

In this narrative review, we aim to focus on the biological activities of the 25(OH)D_3_, providing insight into calcifediol-related rapid non-genomic cell responses and discussing the possible use of this vitamin D metabolite as a medical therapy in individuals at higher risk of hypovitaminosis D.

## 2. Definition of Hypovitaminosis D Status

The general consensus on the definition of optimal serum 25(OH)D_3_ level remains still controversial [25]. This debate is reflected in the recommendations from different European and international agencies, including the Institute of Medicine (IOM), the Endocrine Society (ES), the European Food Safety Authority (EFSA), the European Society of Endocrinology (ESE), the Working Group of the Australian and New Zealand Bone and Mineral Society, and the Scientific Advisory Committee on Nutrition (SACN). According to the IOM guidelines, the minimum recommended circulating level of 25(OH)D_3_ is 20 ng/mL (50 nmol/L), declaring the optimal level of vitamin D status as a serum value of 25(OH)D_3_ greater than 30 ng/mL (75 nmol/L) [26]. The same cut-off values for serum concentrations of 25(OH)D_3_ have been recommended by the EFSA and the Working Group of the Australian and New Zealand Bone and Mineral Society [27,28]. Based on the ES Practice Guidelines [29], vitamin D deficiency has been defined as 25(OH)D_3_ < 30 ng/mL (75 nmol/L), while the most advantageous serum levels for 25(OH)D_3_ were 36–40 ng/mL (90–100 nmol/L). Similarly, the recommended serum level of 25(OH)D_3_ > 30 ng/mL was also proposed by the ESE. The discrepancy between the recommendations of other agencies reflects a lack of standardization in the dosage of 25(OH)D_3_ [30]. In this regard, the ES Practice Guidelines use intact PTH (iPTH) assays, namely the serum level of 25(OH)D_3_ at which iPTH is maximally suppressed, to define the optimal vitamin D status. However, although several studies have found an iPTH mean plateau in individuals with serum 25(OH)D_3_ levels of 30–40 ng/mL, other studies have failed to find a suppression threshold for 25(OH)D_3_ in which iPTH reached a plateau or they found a higher suppression point for 25(OH)D_3_ compared to that recommended (i.e., 10–24.9 ng/mL). Additionally, the relationship between vitamin D status and health outcomes was also investigated in a 2016 report by the SACN Working Group on Vitamin D (available online: https://www.gov.uk/government/publications/sacn-vitamin-d-and-health-report; accessed on 18 April 2023). In their study, the SACN proposed that the rickets risk increased in children and adults with 25(OH)D_3_ levels < 10 ng/mL and between 1.6 and 8 ng/mL, respectively. Furthermore, they suggested that serum 25(OH)D_3_ concentrations below 10 ng/mL were not indicative of disease but were indicative of a higher risk of developing musculoskeletal disorders.

Overall, there is a good but not unanimous agreement that 25(OH)D_3_ values < 20 ng/mL are suboptimal for musculoskeletal health outcomes (i.e., rickets in children, osteomalacia in adults, and secondary hyperparathyroidism), while no serum 25(OH)D_3_ concentration requirements have been established for extraskeletal health outcomes. According to this accepted criterion, approximately 37% of the world’s population suffers from mild vitamin D deficiency (serum 25(OH)D_3_ less than 20 ng/mL), and 7% from severe vitamin D deficiency (serum 25(OH)D_3_ less than 12 ng/mL), which represents a higher risk of developing musculoskeletal disorders [31].

In Table 1, we have summarized the most commonly used definitions of vitamin D status according to the different agencies.

## 3. Vitamin D Deficiency and Related Disorders

Vitamin D deficiency in children is the most common cause of rickets. Persistent vitamin D deficiency or insufficiency in adults leads to decreased bone density, causing osteomalacia, contributing to osteoporosis, and it has been significantly associated with a higher risk of total fracture and hip fractures [32,33]. Conversely, no evidence has been found for an association between serum 25(OH)D_3_ values > 20 ng/mL and bone health benefits in the general population [34,35]. It is well known that vitamin D plays a crucial role in calcium and phosphate homeostasis, impacting the maintenance of physiological PTH levels. By counteracting the eventual excess of PTH, vitamin D supplementation can minimize bone turnover and increase bone mineral density (BMD) at both vertebral and non-vertebral levels. Therefore, vitamin D plays an important role in the prevention and treatment of osteoporosis and fragility fractures. 

In addition, vitamin D deficiency, due to its pleiotropic effects, is associated with proximal muscle weakness and has been identified as a predisposing risk factor for several human diseases, including cardiovascular disease, autoimmune diseases, bacterial, fungus, viral infections, and several types of cancer (i.e., breast, colorectal, and prostate gland) [36]. Vitamin D deficiency appears to be an important risk factor for coronary artery disease (CAD), atrial fibrillation (AF), and heart failure (HF), and a predictor of worse short- and long-term outcomes in all these pathological conditions. However, conflicting results have been reported regarding the effects of vitamin D supplementation in the prevention and treatment of various cardiovascular diseases.

In addition, a consistent association between low 25(OH)D_3_ levels and autoimmune diseases (i.e., multiple sclerosis, rheumatoid arthritis, and type 1 diabetes) has been reported, showing a potent immunomodulatory effect of vitamin D on white blood cells [37,38]. In a recent randomized controlled trial (RCT), vitamin D supplementation, alone or in combination with omega 3 fatty acids, was shown to reduce the incidence of autoimmune disease by 22% [39]; however, data from robust RCTs to confirm these findings are missing so far. 

Over the years, various studies have shown that vitamin D has anticancer properties. Laboratory and animal studies have shown that vitamin D may prevent carcinogenesis and slow tumor progression by inhibiting cell proliferation, promoting cell differentiation, and exerting pro-apoptotic and anti-angiogenetic effects [40]. It has been reported that higher serum levels of 25(OH)D_3_ at diagnosis have been linked to longer survival in cancer patients [41]. A recent review [42] investigating the results of the pivotal RCTs, which addressed the relationship between vitamin D supplementation and the clinical outcomes in colorectal cancer patients, supported the conclusion that even though additional clinical trials are needed to clarify the effect of vitamin D supplementation on the morbidity, mortality, recurrence, and progression of colorectal cancer, lower serum 25(OH)D_3_ levels are inversely associated with colorectal cancer. Moreover, a metanalysis by Voutsadakis [43] showed that low 25(OH)D_3_ levels were associated with breast cancer at diagnosis. Interestingly, a recent study [40] analyzed the effect of vitamin D supplementation on the development of advanced cancer by performing a secondary analysis of the RCTs, showing that supplementation with vitamin D reduced the incidence of metastatic and fatal advanced cancer in the entire cohort, compared to the placebo.

Another non-classical vitamin D action that has gained attention in the context of the COVID-19 pandemic is the therapeutic potential of vitamin D as a major regulator of innate antimicrobial and antiviral responses relative to pathogens. In this regard, pilot randomized clinical studies suggested that achieving serum 25(OH)D_3_ concentrations ≥ 30 ng/mL in COVID-19 patients receiving calcifediol was associated with a faster resolution of symptoms and lower mortality compared to those without calcifediol treatment [44,45,46]. There is also scientific evidence to support the anticancer effects of vitamin D. 

## 4. Treatment of Vitamin D Deficiency

Habit strategies, such as regular sun exposure and increasing the amount of natural foods rich in vitamin D, including dairy products, can help restore proper vitamin D status [47]. It has been observed that 15 min of exposure to sunlight on both sides of the body at noon in summer typically provides 10,000 IU of vitamin D to the systemic circulation in adults. In addition, sun exposure one or two times a week is likely to maintain 25(OH)D_3_ levels within healthy ranges. However, these two strategies are hardly feasible to implement in practical settings because of the problems associated with the UVB-related potential mutagenic risk and the small amount of vitamin D in food. Therefore, vitamin D supplements, especially cholecalciferol, the most commonly used oral supplementation, are very important for improving vitamin D status worldwide [48].

Recently, several studies have examined and compared the effectiveness of vitamin D supplementation with respect to the biological and chemical characteristics and physiopathological aspects of cholecalciferol or calcifediol [6,48,49]. Quesada-Gomez and Roger Bouillon [6] suggested that in certain cases oral calcifediol could be an effective medical option compared to oral cholecalciferol. In particular, the authors emphasized that the medical benefits of calcifediol may be due to the different intestinal absorption mechanisms of the two compounds. Indeed, unlike cholecalciferol, which is absorbed by a complex mechanism through the lymphatic pathway, calcifediol is absorbed from the intestine through the portal circulation. The absorption effect of cholecalciferol in healthy individuals is good but not complete (approximately 79%). In addition, this hydrophobic molecule is poorly absorbed in people with intestinal fat malabsorption. In contrast, the intestinal absorption of calcifediol is approximately 93% in both healthy individuals and individuals with pathological conditions of the small intestine. Such differences in the absorption mechanisms could partially explain the greater overall bioavailability of orally taken calcifediol.

By the overall analysis of studies comparing the oral administration of both a single dose or multiple dosages of cholecalciferol and calcifediol, the authors in [6] found that oral intake of calcifediol more strongly increases circulating 25(OH)D_3_ concentrations than oral cholecalciferol, also presenting a more linear relationship between the orally taken dose and the final serum concentration. This property is due to the fact that calcifediol is much more efficiently absorbed from the gut and is already 25-hydroxylated, thus making it independent of the hepatic 25-hydroxylase activity or the presence of liver diseases or medical therapies that affect the hepatic CYP450 enzyme system. When administered at similar low daily dosages (25 μg/1000 IU), calcifediol was approximately two to five times more potent, with an average of 3.2. The potency of calcifediol was even higher when higher dosage (>2000 IU/day) administrations were compared, ranging from 5.5 to 12 times. 

Another important aspect to consider when comparing the bioavailability of the two metabolites of vitamin D is their different polarity. Calcifediol is a more hydrophilic molecule than cholecalciferol and presents a shorter half-life (approximately two weeks versus two months) [50]. Because of its hydrophobic nature, a large proportion of the cholecalciferol produced in the epidermis is trapped by adipose tissue and gradually released when an active vitamin D metabolite is requested [51,52]. This property is important for raising patients’ compliance and treatment adherence because cholecalciferol can be administrated at high intermittent doses. However, on the other side, due to the high sequestration of cholecalciferol in the adipose tissue, calcifediol could be more available and effective in obese patients and in those individuals who have undergone bariatric surgery, a common cause of severe hypovitaminosis D [49].

## 5. Biological Activity of Calcifediol: Rapid Non-Genomic Responses

The first demonstration of the non-genomic action of steroid hormones was presented in 1942 in a study by Hans Selye, who described that progesterone in addition to its known primary hormonal activity, which occurs after an incubation period of several hours to several days after injection, was able to induce deep and rapid narcosis in mice and rats by peritoneal injection [53]. Subsequent studies by Spach and Streeten showed that aldosterone stimulated sodium exchange in canine erythrocytes within a few minutes after administration, providing new evidence of a non-genomic effect in nucleus-free cells. This fact rules out a priori the possibility of gene transcription and hence genomic action [54]. However, only later studies have shed light on the mechanisms related to the rapid non-genomic action of steroid hormones.

In 1984, Nemere et al. [55] were the first to report the function of 1α,25(OH)_2_D_3_-independent gene transcription and mRNA translation, and found that a rapid unidirectional rise in calcium transport from the lumen to the venous effluent in normal vitamin D-replete chicks was found within 14 min.

Over recent years, other studies have investigated 1α,25(OH)_2_D_3_-related rapid non-genomic actions, with an emphasis on the possible molecules involved and the biological consequences of their activation. Calcitriol has been shown to interact with the membrane isoform of VDR (mVDR) associated with caveolin (CAV1) and the non-classical membrane-associated rapid response steroid 1,25(OH)_2_D_3_-MARRS binding receptor, resulting in the activation of rapid non-genomic effects in target cells [56,57,58]. When calcitriol binds to mVDR and 1,25(OH)_2_D_3_-MARRS, different signaling molecules, such as phospholipase C (PLC), phosphatidylinositol-3 kinase (PI3K), and phospholipase A2 (PLA2), are activated, thereby generating second messenger molecules, such as cyclic adenosine monophosphate (cAMP), phosphatidylinositol (3,4,5)-trisphosphate (PIP3), and calcium, resulting in the downstream activation of protein kinases, such as protein kinase C (PKC), calcium/calmodulin-dependent protein kinase II gamma (CaMKIIG), mitogen-activated protein kinases, and Src [59,60,61,62,63]. In addition, numerous signaling transduction pathways have been demonstrated to be activated upon receptor/vitamin D metabolite binding, including sonic hedgehog (Shh) [64,65,66,67,68,69], Wnt [70,71,72,73], and Notch [74,75,76].

Among the most well-studied effectors of 1,25(OH)_2_D_3_-MARRS, there is the protein disulphide isomerase family A member 3 (Pdia3) [58], whose interaction not only plays a relevant role in one of the most characterized non-genomic actions of 1,25(OH)_2_D_3_ known as transcaltachia, but also suppresses calcium-induced tumor necrosis factor receptors, protects against UV light-induced DNA damage, and increases calcium levels in aortic smooth muscle cells.

For a long time, calcifediol was thought to be only a mild prohormone, a precursor of the active vitamin D; however, it has recently been demonstrated to bind and activate VDR, although to a lesser extent (approximately 50 times lower), compared to the biologically active form of vitamin D, thus exerting antiproliferative activity and gene regulatory functions mediated by the nuclear VDR [20,77,78,79,80].

Likewise, calcifediol could also be supposed to have a rapid non-genomic activity. 

A recent previous study by our research group [81] demonstrated a rapid non-genomic action of calcifediol in the pre-osteoblastic mesenchymal stem cell lines derived from human adipose tissue (hADMSCs) when administered at the high concentration of 10 μM, which resulted in a significant rapid and sustained increase in intracellular calcium concentration.

A study carried out by Asano et al. [82] showed a VDR-independent non-genomic mechanism of action of calcifediol on the regulation of lipid metabolism, exerted by acting as an inhibitor of the sterol regulatory element-binding proteins (SREBPs), and thus inducing proteolytic processing and the ubiquitin-mediated proteasomal degradation of the cleavage-activating protein (SCAP). The results of this study suggest a previously unrecognized molecular mechanism of calcifediol-mediated lipid control, independent of the VDR, which might be useful in the treatment of metabolic diseases.

## 6. Discussion

Mild or severe vitamin D insufficiency is largely prevalent globally, affecting millions of people.

Therefore, vitamin D supplementation is considered a pivotal strategy to restore proper vitamin D status. There is still an ongoing debate about when to start vitamin D supplementation, with values of 25(OH)D_3_ levels ranging from 20 to 30 ng/mL [83].

It is well established that maintaining circulating 25(OH)D_3_ levels between 30 and 50 ng/mL is important for maximizing the benefits and reducing the risks associated with hypovitaminosis, particularly in subjects at higher risk, such as children, the elderly population, and people with minimal sunlight exposure. Recent reviews [31,84,85,86] analyzed the patterns of vitamin D status worldwide to assess differences by region, age, and sex. Interesting discrepancies were observed when the analysis was stratified by geographical regions. In particular, in the Asia/Pacific region, lower levels of 25(OH)D_3_ were observed in the Chinese children/adolescent groups compared to older groups because of their limited sun exposure and lower intake of calcium. On the contrary, in the Middle East/Africa region, better vitamin D status was found in the younger population because adolescents spend more time outdoors compared to the older groups. Overall, a better vitamin D status was observed in North America, probably due to the routine consumption of fortified foods with vitamin D. Within Europe, the highest 25(OH)D_3_ values were found in northern Europe compared to the eastern and southern European countries, a finding that could be explained either by differences in skin pigmentation or oily fish-based diets. Vitamin D deficiency tends to be most common among residents of industrialized countries who spend more time indoors. In general, vitamin D deficiency is more common in older adults, due to inadequate sunshine exposure and decreased hydroxylases activity, and it is more pronounced in institutionalized and housebound geriatric patients.

**Figure 2:**
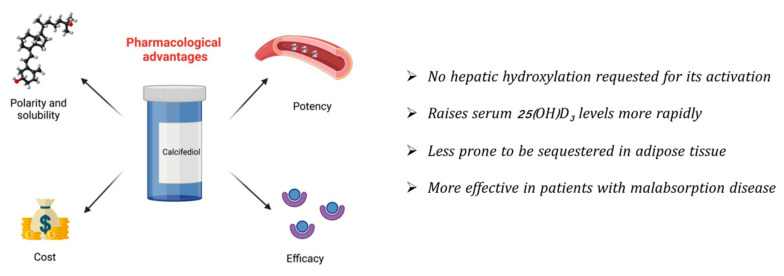
Scheme of potential advantages of calcifediol as a vitamin D supplement compared with cholecalciferol for the treatment of vitamin D deficiency status.

Most of the evidence from registered clinical trials conducted on patients with low BMD is related to cholecalciferol. Recently, however, calcifediol has also been considered as an oral vitamin D supplement to bring benefits to people at higher risk of hypovitaminosis D. Based on the previous results, oral calcifediol supplementation could have more advantages compared to cholecalciferol (Figure 2).

First, calcifediol has been shown to rapidly increase circulating 25(OH)D_3_ concentrations and it is more potent compared with cholecalciferol, which means that not only can it correct vitamin D deficiency more promptly but it can also be used at lower doses. Second, calcifediol is highly polar and soluble, making it not only more difficult to retain in fatty tissue but also to have greater efficacy in intestinal absorption compared to cholecalciferol. As a result, calcifediol administration could be especially useful for obese individuals or those suffering from malabsorption syndrome. In addition, because calcifediol does not require hydroxylation in the liver, it exhibits a more linear concentration–response curve than cholecalciferol, making it more suitable for the treatment of people with liver disease and those taking drugs that inhibit the action of CYP450 enzymes.

In this regard, a study by Pérez-Castrillón et al. [87] revealed that calcifediol is a safe and effective treatment to reach the optimal 25(OH)D_3_ concentration in a cohort composed of 298 postmenopausal vitamin-D-deficient patients (osteoporotic and non-osteoporotic). Specifically, they found that calcifediol is faster and more powerful compared to cholecalciferol in achieving serum 25(OH)D_3_ levels above 30 ng/mL after 4 months of treatment, suggesting that this supplement may be an effective treatment strategy for vitamin D deficiency.

According to the latest updated publication of the European Society for Clinical and Economic Aspects of Osteoporosis, Osteoarthritis and Musculoskeletal Diseases (ESCEO) [88], calcifediol should be recommended as the form of vitamin D of choice when a rapid improvement of vitamin D status is required, such as in osteomalacia, non-dialysis chronic kidney disease (CKD), obese and malabsorptive patients, men with hypogonadism, and patients with liver disease.

One of the most important issues that supports the use of cholecalciferol supplementation is that the vitamin D recommended dosage is provided by international units (IU). However, it should be emphasized that the gold standard of the equivalence between the molecular mass expressed in micrograms of vitamin D and IU is set for cholecalciferol. In particular, 1 IU is equivalent, in terms of vitamin D biological activity, to that provided with 0.025 μg of cholecalciferol, but the same translation is not possible for calcifediol [89]. Therefore, it is not possible to guarantee an adequate estimate of the IU amount when taking calcifediol. Although some clinical trial data have attempted to address this issue, there is currently no way to provide an accurate μg to μg calculation between cholecalciferol and calcifediol.

Some common doses of calcifediol used in clinical practice have been shown to elevate serum 25(OH)D_3_ levels above the physiological level, especially when taken intermittently. There is scientific evidence that such excessive values of 25(OH)D_3_ could impact health and bone metabolism homeostasis negatively [90,91,92]. Toxicity associated with vitamin D overdose could be attributed both to the responsibility of health care providers (i.e., medical prescription or dispensing errors) or to errors in medicine self-administration by the patient. As opposed to calcifediol, supplementation with cholecalciferol may reduce the likelihood that 25(OH)D_3_ levels will rise above the physiological cut-off. According to these data, ensuring that 25(OH)D_3_ levels do not exceed 50 ng/mL may be important to prevent potential health risks, as recommended by the IOM. Therefore, it could be extremely important to implement in clinical routine the monitoring of 25(OH)D_3_ levels, especially in patients treated with calcifediol.

In relation to cost, with the assumption of a higher pharmacologic activity of 25(OH)D_3_ compared to oral cholecalciferol, the direct precursor of biologically active vitamin D would cost approximately about half as much as cholecalciferol (according to market prices in Italy in 2023).

## 7. Conclusions

Given that vitamin D deficiency represents a common health problem worldwide, achieving and maintaining adequate serum 25(OH)D_3_ levels becomes crucial to avoid detrimental effects not only on musculoskeletal health but potentially on a wide range of acute and chronic diseases. Despite the lack of data on the cost effectiveness of vitamin D supplementation in the general population, there is no doubt that vitamin D analog supplements should be recommended for those at high risk for vitamin D deficiency. There is no current consensus on the most appropriate treatment scheme for correcting vitamin D deficiency; therefore, diverse international agencies and societies have not only developed guidelines for the optimal serum 25(OH)D_3_ levels but also for appropriate vitamin D supplementation. Bone-centric guidelines recommend vitamin D doses of 400–800 IU/day depending on age. As opposed to these recommendations, guidelines focusing on the multifaceted effects of vitamin D propose daily vitamin D supplementation doses ranging from 400 to 2000 IU/day depending on diverse factors (i.e., age, ethnicity, body mass index, and clinical context).

Compared with cholecalciferol, calcifediol is more hydrophilic, has a shorter half-life, does not require hepatic 25-hydroxylation for its activation, has a more linear and predictable dose–response relationship, and increases serum 25(OH)D_3_ levels more rapidly. These properties could point to a decision in favor of taking calcifediol. Overall, its use could be more advantageous in people affected by malabsorption syndromes or liver failure and in vitamin D deficiency conditions that require a faster increase in serum 25(OH)D_3_ levels, such as for COVID-19.

**Figure 3:**
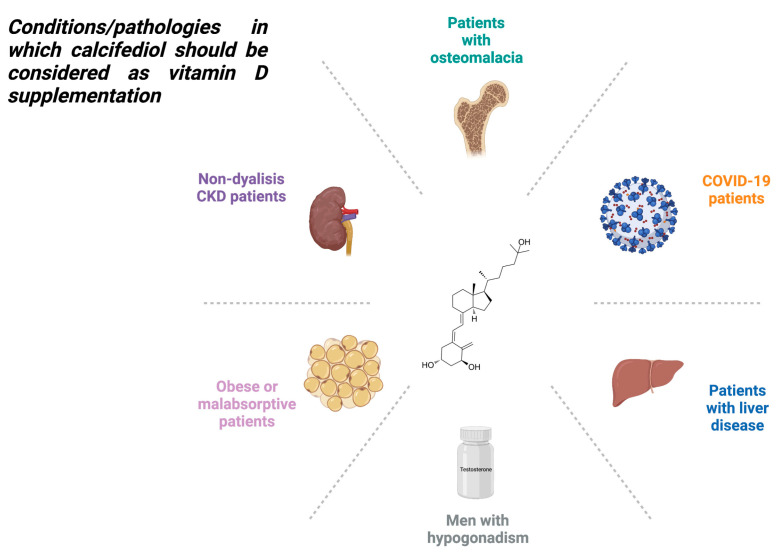
Conditions in which the administration of calcifediol could be preferable as a vitamin D supplement form.

In Figure 3, we summarize the most common disorders for which calcifediol might be preferable to cholecalciferol administration.

## Figures and Tables

**Table 1:** Different thresholds of serum 25(OH)D_3_ concentrations for the definition of vitamin D deficiency and sufficiency proposed by diverse international agencies and societies.

Serum 25(OH)D_3_ Levels (ng/mL)	IOM	ES	EFSA	Working Group of the Australian and New Zealand Bone and Mineral Society	SACN	ESE
<10	Vitamin D deficiency	Vitamin D deficiency		<5 ng/mL (severe vitamin D deficiency) 5–11.6 ng/mL (moderate vitamin D deficiency)	Vitamin D deficiency	Vitamin D deficiency
10–20	Vitamin D insufficiency	Vitamin D deficiency		12–19.6 (mild vitamin D deficiency)	Sufficient	Vitamin D deficiency
20–30	Sufficient	Vitamin D insufficiency	Sufficient	Sufficient	Sufficient	Vitamin D insufficiency
>30	Sufficient	Sufficient		Sufficient	Sufficient	Sufficient

Abbreviations: IOM, Institute of Medicine; ES, Endocrine Society; EFSA, European Food Safety Authority; SACN, Scientific Advisory Committee on Nutrition; ESE, European Society of Endocrinology.

## Data Availability

Not applicable.
